# Hypofractionated Stereotactic Radiotherapy for Non-breast or Prostate Cancer Oligometastases: A Tail of Survival Beyond 10 Years

**DOI:** 10.3389/fonc.2019.00111

**Published:** 2019-02-27

**Authors:** Khush S. Aujla, Alan W. Katz, Deepinder P. Singh, Paul Okunieff, Michael T. Milano

**Affiliations:** ^1^Department of Radiation Oncology, University of Rochester Medical Center, Rochester, NY, United States; ^2^Department of Radiation Oncology, University of Florida College of Medicine, Gainesville, FL, United States

**Keywords:** oligometastases, radiotherapy, metastases, stereotactic radiation, survival

## Abstract

**Purpose and Objective(s):** We sought to analyze the long-term follow-up of patients treated with hypofractionated, stereotactic radiotherapy (HSRT) for oligometastases from malignancies other than breast or prostate cancer.

**Materials and Methods:** From 2001 to 2006, 82 cancer patients with 1–5 radiographically apparent metastatic lesions (in 1–3 organs) from primary sites other than breast or prostate cancer, were enrolled on a prospective study of HSRT. Freedom from widespread metastasis (FFWM) was defined from date of enrollment until death, an event (i.e., widespread distant metastasis not amenable to local therapy), or last radiographic study. Local recurrence was scored as an event if pathologically confirmed or if a treated lesion increased by ≥20% using RECIST criteria. Prognostic variables were assessed using Cox regression analysis.

**Results:** The mean age was 61 ± 11 years, with a male to female ratio of 46:36. The most common metastatic sites were liver (50%), lung (48%), thoracic lymph nodes (18%), and bone (5%). Sixty-one patients (74%) had 1 involved organ and 18 (22%) had 1 lesion treated. The preferred dose-fractionation scheduled was 50 Gy in 10 fractions (52 patients). The median follow-up was 1.7 years. Eleven patients lived >5 years, and 6 lived >10 years. The 5-year OS, PFS, FFWM, and LC rates were 13.4, 7.3, 18.3, and 63.4%, and the 10-years OS, PFS, FFWM, and patient LC rates were 7.3, 6.1, 13.4, and 62.2%, respectively. A greater net gross tumor volume (GTV) was significantly adverse for OS (*p* < 0.01) and LC (*p* < 0.01). For FFWM, net GTV was not a significant factor (*p* = 0.14). Four patients remain alive at >13 years from enrollment and treatment, without evidence of active disease.

**Conclusion:** A small subset of select non-breast, non-prostate cancer patients with limited metastasis treated with HSRT are long-term survivors. Net GTV is a significant factor for tumor control and survival. Further research is needed to help better select patients most likely to benefit from local therapy for metastatic disease.

## Introduction

In 1995 Hellman and Weichselbaum hypothesized a “clinical significant state of oligometastases,” in which metastases limited in number and extent represented a relatively indolent disease state before reaching full metastatic potential (1). It has since been postulated that oligometastases may possess unique genetic characteristics, and are potentially amenable to definitive, metastases-directed (i.e., surgery or ablative) treatment (2). The interest in metastasis directed therapy dates back decades (3, 4). In 1968 Rubin questioned “Are metastases curable?” in a JAMA editorial (5) in addition to writing a book “Solitary Metastasis” (3) in which localized therapies were discussed. In 1983, Peters, Milas, and Fletcher explored the concept of systemic therapy to sterilize occult metastatic disease, followed by radiation therapy to overt sites of disease as a curative treatment (6).

Systemic therapy remains the standard of care for metastatic disease. As the efficacy of systemic therapy continues to improve with the development of novel agents, durable tumor control becomes more important in patients with limited metastatic disease. Recent advances in radiographic and functional imaging, ablative techniques, and radiotherapy have again made the hypothesis of metastasis-directed therapy for oligometastases more compelling. Hypofractionated, stereotactic radiotherapy (HSRT), or stereotactic body radiation therapy (SBRT) if delivered in up to 5 fractions, is an advanced treatment technique enabling the delivery of high biologically effective doses to the target while limiting normal tissue volume receiving therapeutic doses (7). The high fractional doses of radiation are postulated to have the ability to overcome intra-tumor regional hypoxia as well as potentially stimulate an immune response (8), and have become an acceptable treatment for oligometastases.

There is a growing body of evidence supporting the use of radical irradiation for oligometastases (9, 10). The recently reported multi-national SABR-COMET, randomized 99 patients in a 1 to 2 ratio between standard of care ± SBRT (*n* = 65 non-breast, non-prostate cancer) with 1–5 oligometastases. SBRT significantly increased the median progression-free survival (PFS; 12 vs. 6 months *p* = 0.001); the median overall survival (OS) difference (41 vs. 28 months, *p* = 0.09) met the study's randomized phase II endpoint of *p* < 0.20 (11).

While breast and prostate cancer tend to have better outcomes, both overall and in the oligometastatic setting (12), several studies have shown potential benefits for oligometastatic therapy for other primaries. Gomez et al. randomized stage IV NSCLC oligometastatic patients with three or fewer metastatic lesions after first line systemic therapy to either local consolidative therapy with or without subsequent maintenance treatment, or to maintenance treatment alone in a randomized phase II trial. The study was terminated early after 49 patients were randomized, with the interim analyses showing a significant improvement in median PFS in the local consolidative group (11.9 vs. 3.9 months) (13). Iyengar et al. conducted a similar trial assessing consolidative radiotherapy in limited metastatic (primary plus up to 5 metastatic sites) NSCLC, and also stopped early after an interim analysis showed improved PFS for local consolidative therapy (9.7 vs. 3.5 months) (14). Studies of oligometastatic colorectal patients have long shown a survival benefit with resection of limited lung or liver metastases (15–17). There are now multiple series showing excellent outcomes with metastasis-directed therapy in lung, liver, adrenal, lymph nodes, and bone oligometastases (18–23), (23).

Long-term (10+ year) data on metastasis-directed radiotherapy for oligometastatic cancer are lacking. There are even more limited data for non-breast, non-prostate metastatic primaries. We previously published the survival and tumor control outcomes of 121 patients with five or fewer radiographically apparent metastases from any primary site (including 39 breast cancer patients, and no prostate cancer patients), metastatic to any organ, treated with HSRT with curative intent (24). We sought to analyze the 10-year outcomes of the non-breast, non-prostate oligometastatic patients treated with HSRT on a prospective Phase II protocol in an effort to better understand long-term outcomes and factors that may impact these outcomes.

## Methods And Materials

Between February 2001 and December 2006, 82 patients with one to five radiographically apparent metastatic lesions were enrolled on a University of Rochester Medical Center (URMC) prospective pilot study, using HSRT to treat limited oligometastatic disease (25). The URMC research subjects review board approved the study, and all patients provided written informed consent. The eligibility requirements included age ≥18 years, Karnofsky performance status (KPS) ≥70, and one to five extra-cranial metastases. Prior treatment of metastatic tumor (including radiation or surgery) did not exclude patients from the study unless the treating physician determined that radiation could not be delivered safely. Prior chemotherapy for metastatic disease was allowed. Four patients (each with fewer than five total metastases) also had brain metastases (six lesions among 4 patients) treated with single-fraction radiosurgery. The patients who experienced local recurrence after HSRT in one or more sites, or who developed additional metastatic disease, were allowed to undergo additional courses of HSRT (26). The net GTV represented the sum of each lesion's GTVs according to the contoured volumes on the planning computed tomography scan. The net GTV was calculated at SBRT planning; thus, previously resected metastases were not included in the net GTV. Likewise, changes in the tumor volume resulting from previous systemic therapy were not accounted for. The net GTV did not include oligometastases that developed, and were subsequently treated, after completion of the initial protocol HSRT.

### HSRT Technique

The HSRT technique has been discussed in greater detail in previous publications (27–29) and briefly summarized here. During initial simulation and with all treatments, the patients were immobilized with a vacuum cushion, and the treatment setup was reproduced using a relaxed end-expiratory breath hold technique and the Novalis ExacTrac® patient positioning platform (BrainLAB AG, Heimstetten, Germany). Treatment planning was performed using the BrainSCAN system (BrainLAB AG). The PTV was generated with a minimal GTV expansion of 10 mm in the craniocaudal direction and 7 mm in other directions. Treatment was prescribed to the 100% isodose line, and the PTV was covered by the 80% isodose line. HSRT was delivered using conformal arcs or multiple fixed coplanar beams, shaped with multileaf collimators. The protocol described a range of recommended prescribed doses for 3, 4, 5, 6, 8, and 10 Gy fractional doses(as described in detail previously, but allowed the treating physician discretion, in order to adhere to protocol mandated normal tissue dose constraints (30). The required normal tissue dose-volume constraints have been reported in previous publications (8, 27, 28). Because of various dose-fractionation schedules used, we also analyzed biologically equivalent dose (BED), using an assumed alpha-beta ratio of 10 Gy. Most (63%) of the 202 non-brain lesions were treated to 50 Gy in 10 fractions.

### Endpoints

Widespread distant metastases are defined as distant progression not amenable (at the discretion of the treating physician) to resection or locally ablative therapy (i.e., SBRT, HSRT, stereotactic radiosurgery, radiofrequency ablation, and embolization) due to the bulk and/or number (generally more than 3) of metastases. The freedom from widespread distant metastasis (FFWM) and OS rates were calculated using Kaplan-Meier actuarial survival analyses. OS was defined from the date of enrollment until death or the last follow-up visit, and FFWM was defined from the date of enrollment until death, an event (widespread distant progression), or the last radiographic study. Lesion local recurrence was scored as an event if any treated lesion increased by ≥20%, using the Response Evaluation Criteria in Solid Tumors criteria (31), or local recurrence was confirmed pathologically. The treating physician would generally opt to follow with serial imaging, or obtain PET imaging (commissioned in 2005) if there was concern about post-radiation changes mimicking progression. Stata version 15.1 (StataCorp, College Station, TX), was used for all data analysis.

## Results

### Patient Characteristics

The patient and tumor characteristics are summarized in Table 1. Twenty-four patients presented with metastatic disease during their initial diagnostic workup for cancer. For the remaining fifty-nine patients, the interval between initial diagnosis and metastasis was from 3 to 98 months (median 16 months). Fifty patients had a >6 month interval between initial diagnosis and metastatic diagnosis. The patients were generally referred for radiation if they were not candidate for, or declined, systemic therapy (21 patients); for disease progression after receiving systemic therapy (20 patients); after experiencing a clinical response or stable disease after systemic therapy (and therefore referred for consolidative HSRT) (20 patients); for local therapy of new limited metastasis (in conjunction with systemic therapy starting just before or after HSRT) (14 patients); or for growing metastases occurring >6 months after completing systemic therapy (7 patients). The median time from metastasis diagnosis to enrollment was 6.5 months. There were 61 patients that underwent systemic therapy at some point after metastases diagnosis, 40 of those patients underwent systemic treatment prior to HSRT. No patient received immunotherapy. There was radiographic progression after systemic therapy in 20/40 patients, and stable or regressive disease in 20/40 patients. The majority of patients were treated with 10 fractions to a total dose of 50 Gy (52 patients), with 58 patients getting a biological equivalent dose of 75 Gy or greater.

**Table 1:** Patient Characteristics at initial presentation of oligometastatic disease.

**Characteristics**	**No. of patients (%)**	**Characteristic**	**No of patients (%)**
Total no. of patients	82	Primary Histology	
No. alive at last follow up	4	Adenocarcinoma	50 (61%)
No. with no evidence of disease	4	Other	9 (11%)
Age, y		Squamous Cell Carcinoma	7 (9%)
Median (range)	61(41-88)	Sarcoma	7 (9%)
Mean±SD	61 ± 11	Carcinoid	3 (4%)
Male/Female	46/36	Hepatocellular Carcinoma	3 (4%)
Primary cancer		Renal Cell Carcinoma	3 (4%)
Colorectal	31 (38%)	Sites involved with metastatic disease	
Lung, head and neck, or esophagus	23 (28%)	Lung	39 (48%)
Liver, Pancreas	9 (%)	Thoracic lymph node	15 (18%)
Other	9 (11%)	Liver	41 (50%)
Sarcoma	7 (9%)	Pelvic or abdominal lymph node	2 (2%)
Renal	3 (4%)	Brain	4 (5%)
Sum of GTVs ml		Adrenal Glands	4 (5%)
Median (range)	32 (0.3–422)	Bone	4 (5%)
Mean ± SD	55 ± 8	No. of oligometastatic lesions	
Prior curative-intent local therapy	29 (35%)	1	22
Previously had > 5 metastatic lesions	16 (20%)	2	20
Reason for Treatment (Rx)		3	22
No systemic Rx for metastasis	21 (26%)	4	10
Disease Progression after systemic Rx	20 (24%)	5	8
CR/PR/SD after systemic Rx	20 (24%)	No. of involved organs	
New Limited metastasis	14 (17%)	1	61
Growing metastasis, >6 months after systemic Rx	7 (9%)	≥2	21

There were twenty-nine patients who underwent curative intent local therapy for metastases prior to enrollment including; 14 colon, 6 sarcoma, 5 lung, 2 utero-cervical, 1 parotid, and 1 ovarian primary cancer. Sixteen patients were diagnosed with ≥ 5 metastatic lesions at some point before enrollment. These patients were treated with either systemic therapy or radiation, which ultimately resulted in ≤ 5 detectable metastases at the time of enrollment. Among 82 patients, there were 108 organs involved by metastases. Sixty-one patients (74%) had 1 involved organ and 18 (22%) had 1 lesion treated. The most common site for metastases was the liver (50%) with 21/41 of the metastases arising from a colon primary. The next most common sites involved were lung (48%) and thoracic lymph nodes (18%). Fourteen of the fifteen patients with thoracic lymph nodes metastasis also had lung metastases (Table 1).

### Toxicity of HSRT

No patient experienced Grade 4–5 toxicity, and only 1 patient experienced Grade 3 toxicity of non-malignant pleural and pericardial effusion while undergoing liver HSRT as described previously (25). Lower grade toxicities were also described previously (25). No additional toxicity was reported in the subsequent follow-up period.

### Follow-Up Duration

Follow–up ranged from 0.3 to 15 years (median 1.7). Eleven patients lived ≥5 years, the duration ranged from 5.5 to 15 years (median 10.8). Four patients were alive at the last follow up with no evidence of disease (median 13.4). There are seven patients deceased with ≥5 years survival (median 5.8), and two patients deceased with ≥10 years survival (median 11.1). For all patients who died, survival ranged from 0.3 to 11.6 years (median 1.6).

### Survival Outcomes

**Figure 1:**
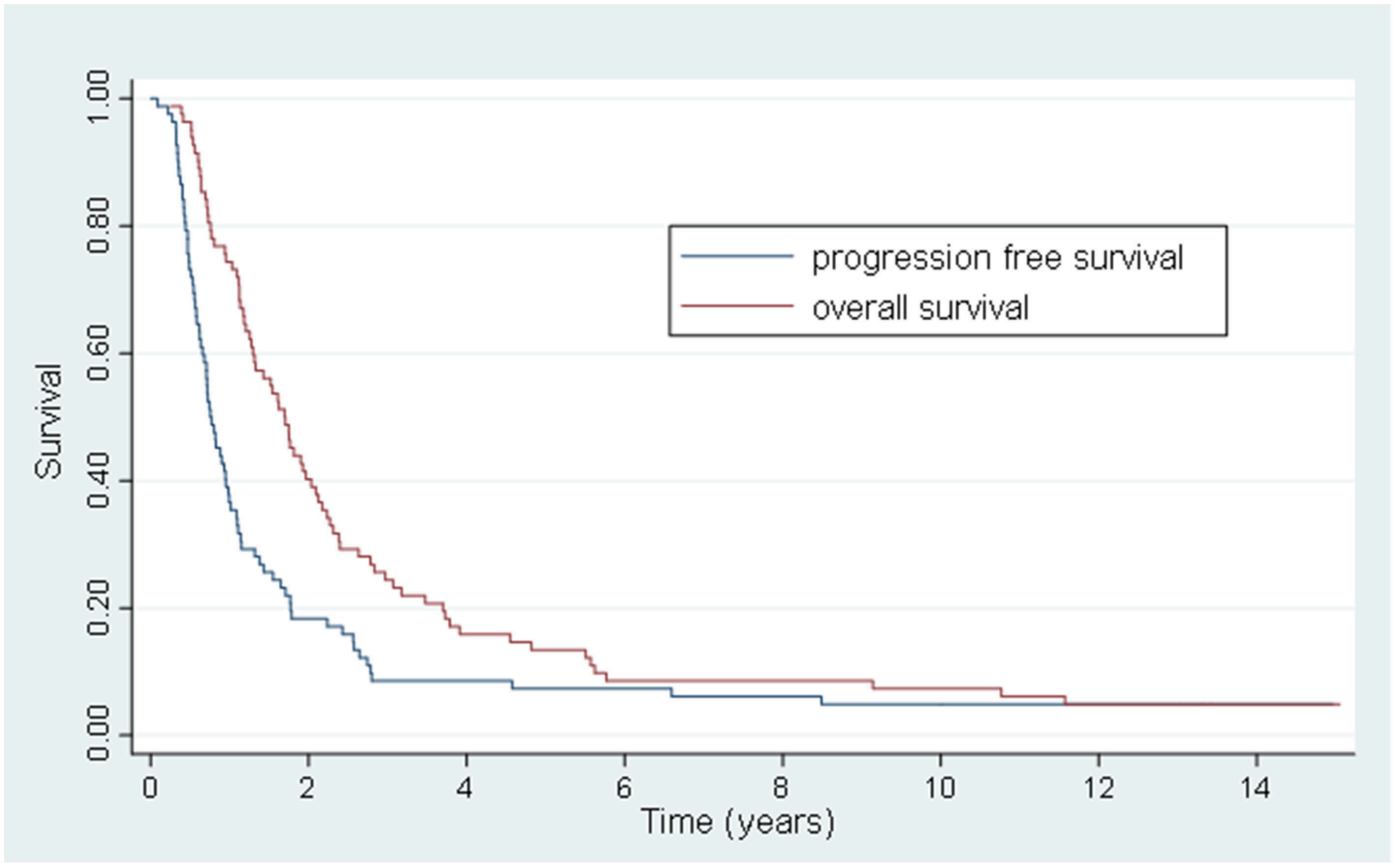
Kaplan-Meier actuarial overall and progression-free survival.

**Figure 2:**
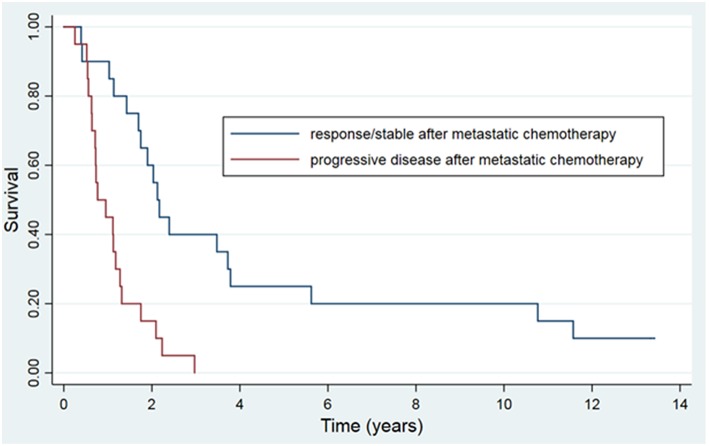
Kaplan-Meier actuarial overall survival, grouped by response to systemic therapy prior to HSRT.

The 5 and 10-year OS rate was 13.4 and 7.3% and the 5 and 10-year PFS rate was 7.3 and 6.1%, respectively (Figure 1). For the 20 patients that were treated with HSRT after progression of lesions after systemic therapy vs. the 20 patients who had stable or regressive disease, the mean survival was 1.1 years vs. 4.2 years (*p* < 0.01) and the mean PFS was 0.6 years vs. 2.6 years (*p* < 0.01) (Figure 2). The characteristics of long-term survivors (≥ 5 years) are shown in Table 2. Ten out of the eleven patients underwent systemic therapy, and 5 out of the 11 had systemic treatment prior to HSRT with stable or good response to chemotherapy. Four patients had prior curative intent local therapy, and 2 patients initially presented with >5 distant metastasis. There were no long-term survivors with more than 2 organs initially involved. At 10 years there were 6 patients that went on to develop either local (6) or distant (5) progression of disease with a PFS range of 1.7–9.3 years (median 4.8 years). There were 6 patients with survival ≥10 years from treatment, with 1 out of the 6 developing new liver metastasis at 9.4 years after initial treatment, and was retreated with local-directed therapy.

**Table 2:** Characteristics of long-term (≥ 5 years) survivors.

**Characteristics**	**No. of patients (%)**	**Characteristic**	**No of patients (%)**
Number of patients	11	Number of involved organs	
Alive at last follow up	4	1	8 (72%)
Follow up of living patients (years)	13.3–15.1 (median 13.4)	2	3 (27%)
Deceased with survival ≥5years	7	Primary Histology	
Survival (years)	5.5–11.5 (median 5.8)	Adenocarcinoma	5 (45%)
Age (years)	57 (49–77)	Squamous Cell Carcinoma	1 (9%)
		Other	5 (45%)^**^
**PRIMARY CANCER**			
Colorectal	3 (27%)	Sum of GTVs (ml)	4-239 (median 15)
Lung, head and neck, or esophagus	2 (18%)	No. of involved organs	
Other	6 (54%)^**^	1	2 (18%)
		2–3	6 (54%)
Initial sites involved with metastases		4–5	3 (27%)
Lung	5 (45%)		
Thoracic lymph node	2 (18%)	Additional therapy for metastases	7 (63%)
Liver	6 (54%)	Local therapy for local recurrence	2 (18%)
Bone	1 (9%)	Local therapy for new oligometastases(es)	6 (46%)

** *Other primary cancers/histologic types included sarcoma (n = 7), pancreas (n Z 4), hepatocellular (n Z 3), carcinoid (n = 3), urinary bladder (n = 3), renal (n Z 3), adrenocortical (n = 1), ovarian (n = 1), endometrial (n = 1), endocervical (n = 1), and melanoma (n = 1)*.

Among 78 deaths, 67 occurred in patients who had developed widespread distant metastases, which was likely the cause of, or major contributor to, death in these patients. One >10-year survivor died from progression of a second primary lung cancer. One patient died at 3 months, with evidence of local progression of liver disease. Three died from other causes, and 6 died from unknown causes (though most were likely from cancer progression). As most deaths were cancer-related, and the cause of death not available for all patients, cancer-specific survival was not analyzed.

As expected, having widespread distant recurrence was a strong predictor for worse overall survival (*p* < 0.01, HR 3.41), but local recurrence was not (*p* = 0.59). Fifty patients had a >6 month gap between initial diagnosis and diagnosis of metastasis, but the time between diagnosis and metastasis did not predict for overall survival (*p* = 0.15). The hypothesis-generating univariate and multivariate analysis of other potential prognostic variables for OS and PFS are listed in Table 3. The net GTV in cm^3^ (cc) was the only analyzed variable significant for OS (*p* < 0.01) and PFS (*p* < 0.01). We categorized each patient's net GTV in bins of 10 cm^3^ to better characterize the significance. For OS and PFS, every increase of 10 cc in net GTV was predictive of a 4% increase in risk of death or progression. This variable was consistently significant for both survival outcomes on MV analysis. In contrast the total number of lesions treated, the site of metastasis, or the number of involved organs did not prove to be significant. On univariate analysis a BED of 75 Gy or greater was borderline significant, though not significant with multivariate analysis.

**Table 3:** Univariate and multivariate analyses of prognostic factors for overall survival and progression free survival.

**Variable**	**OS**	**PFS**
Age (y) (UVA Cox)	0.20	0.76
**PRIMARY CANCER (UVA LOG RANK)**		
Colorectal, *p*	0.71	0.31
Lung, head/neck, esophagus, *p*	0.28	0.49
**SITE INVOLVED (UVA LOG RANK)**		
Lung, *p*	0.97	0.66
Thoracic lymph nodes, *p*	0.77	0.95
Liver, *p*	0.85	0.39
Oligometastatic lesions treated UVA (Cox), *p*	0.77	0.91
involved organs (1 vs. 2-3) UVA (Cox)*, p*	0.53	0.77
History of >5 metastases prior to enrollment (UVA Cox)	0.35	0.59
Systemic therapy for metastasis (UVA Cox)	0.20	0.25
BED 75 Gy or greater (UVA Cox)	0.10	0.14
History of prior curative local treatment (UVA Cox)	0.81	0.65
**SUM OF GTV (CM**^**3**^**)**		
UVA (Cox), *p*	<0.01	0.01
UVA HR (95% CI) per 10 cm^3^	1.04 (1.014–1.075)	1.04 (1.009–1.07)
MVA (Cox), *p*	<0.01	0.03
MVA HR (95% CI) per 10 cm^3^	1.05 (1.014–1.099)	1.04 (1.005–1.09)

### Lesion Local and Distant Control

**Figure 3:**
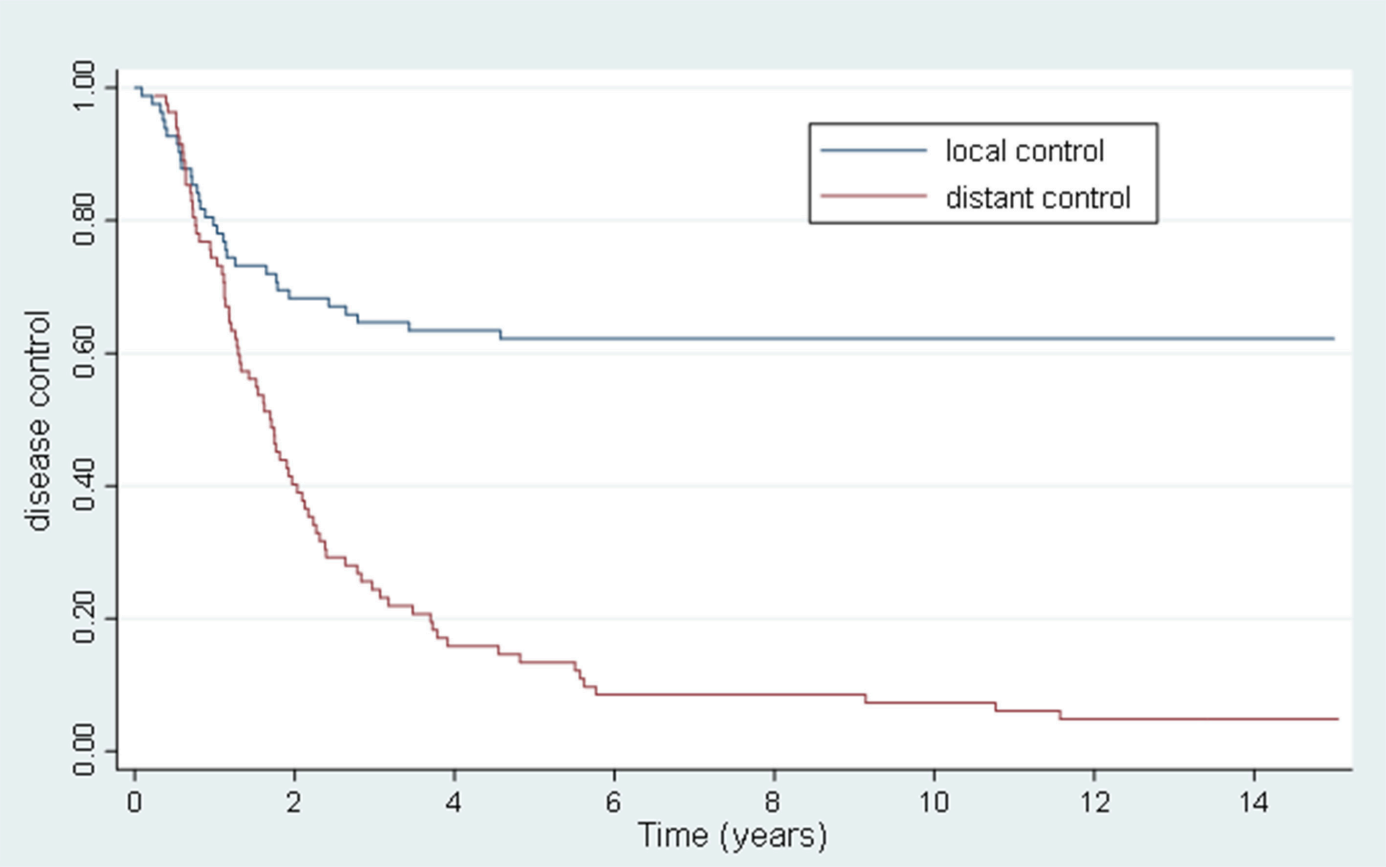
Kaplan-Meier actuarial local (treated-metastasis) and distant control.

The 5 and 10 year local control (LC) was 63.4 and 62.2%, respectively, and the 5 and 10 year FFWM was 18.3 and 13.4%, respectively. Figure 3 summarizes the long-term rate of disease control. The median time to local recurrence was 1.2 years. In comparison there were 69 patients that had distant recurrence also with a median time of 1.2 years. Forty-nine of the patients with distant recurrence had received some form of systemic therapy. The hypothesis-generating univariate and multivariate analyses of potential prognostic factors for disease control are listed in Table 4. Net GTV was shown to be highly significant predictor for local control (*p* < 0.01) with a HR of 1.09 for every 10 cc of net GTV for both univariate and multivariate analyses. A BED of 75 Gy or greater was significant on univariate analysis (HR = 0.37), though was not significant on multivariate analyses. There were 11/39 recurrences for patients with oligometastases lesions treated in the lung, and 20/41 recurrences for patients with liver lesions (*p* = 0.16 and *p* = 0.12, respectively). Nine out of the 31 recurrences had previously undergone prior curative intent local therapy, and eight patients initially had more than 5 metastasis (NS). The number of lesions treated also did not predict for overall survival. Systemic therapy during metastases diagnosis was the only variable that approached significance for FFWM on MV analyses (*p* = 0.09). Out of the 69 patients who had distant recurrence, 20 were unable to get systemic treatment. On UV and MV for both LC and FFWM the primary site of disease (colorectal, lung/esophagus/head and neck) were not significant.

**Table 4:** Univariate and multivariate analyses of prognostic factors for local control and freedom from distant progression.

**Variable**	**LC**	**FFWM**
Age (y) (UVA Cox)	0.94	0.81
**PRIMARY CANCER (UVA LOG RANK)**		
Colorectal, *p*	0.38	0.30
Lung, head/neck, esophagus, *p*	0.20	0.50
**SITE INVOLVED (UVA LOG RANK)**		
Lung, *p*	0.16	0.48
Thoracic lymph nodes, *p*	0.52	0.91
Liver *p*	0.12	0.25
Number of oligometastatic lesions treated UVA (Cox), *p*	0.90	
Involved organs (1 vs. 2-3) UVA (Cox)*, p*	0.78	
History of >5 metastases prior to enrollment (UVA Cox), *p*	0.59	0.98
Systemic therapy for metastasis (UVA Cox), *p*	0.25	0.15
MVA (Cox), *p*	0.45	0.09
**BED≥75 GY**		
BED≥75 Gy (UVA Cox), *p*	<0.01	0.15
UVA HR (95% CI)	0.37 (0.18-0.76)	
MVA (Cox), *p*	0.34	0.39
MVA HR (95% CI)	0.61 (0.23-1.68)	
History of prior curative local treatment (UVA Cox)	0.25	0.97
**SUM OF GTV (CM**^**3**^**)**		
UVA (Cox), *p*	<0.01	0.14
UVA HR (95% CI) per 10 cm^3^	1.09(1.05–1.13)	
MVA (Cox), *p*	<0.01	0.17
MVA HR (95% CI)	1.10 (1.04–1.16)/10cm3	

## Discussion

To our knowledge, this study represents the longest follow-up after HSRT for oligometastatic cancer. Specifically, in our study of 82 oligometastatic patients from “less-favorable” primaries (non-breast, non-prostate), we demonstrate that roughly 13% experience long-term (>5-year) survival, with six patients alive past 10 years. At last follow-up, there were 4 patients alive without any evidence of disease. Patients with a lower gross tumor burden fared significantly better in terms of OS, PFS, and lesion LC; however the tumor burden was not significant in predicting for FFWM. Patients whose metastatic lesions were treated with systemic treatment, before HSRT, and had demonstrated radiographic progression after systemic therapy fared significantly worse than patients with stable or regressing disease.

In comparison to the 5 year OS (46%) and PFS (16%) presented for the local ablation arm of SABR-COMET, our 5 year OS (13.4%) and PFS (7.3%) is much lower. One possible explanation is the differences in the site of original primary tumor. Out of the 66 patients treated on the SABR-Arm, 13 (19.7%) had breast cancer primaries and 14 (21.2%) had prostate cancer primaries. The investigators addressed a discrepancy of number of prostate cancers between the control and SABR-Arm, by performing a sensitivity analysis that showed an expected improvement in PFS for prostate primary vs. others. Despite removing these favorable patients, SABR-COMET still showed improved PFS for local ablative therapy (13). There were no specific results presented for the breast cancer patients, but we have previously reported long-term outcomes (4 and 6 years) for both breast and the non-breast oligometastatic groups on this prospective study. Breast cancer patients fared significantly better in terms of OS, LC, and FFWM in comparison to all other primaries (24). The long-term overall survival for breast patients at 10 years was 31%; with osseous-only oligometastases doing significantly better than non-osseous sites (*p* = 0.002) (32). Yet, despite having “less favorable” primaries, our patient cohort showed potential for long-term survival.

Local recurrence was not significantly associated with OS, likely reflecting potential salvage of local recurrence with surgery or re-irradiation. As expected, FFWM was a strong predictor for OS. The only other factor that significantly predicted for OS and PFS was net GTV, with a 4% increase in risk for every 10 cc of tumor burden. The number of metastatic lesions, potentially another parameter of tumor burden, was not a significant factor, as seen in other studies (33).

The predictive role of tumor burden is consistent with the important characteristics for metastasis as first demonstrated by RTOG 9508, which analyzed 1 vs. more than one metastasis (34). In a recent review, Palma, Louie and Rodrigues further described the four key prognostic variables, which they term “four aces,” for patients in the setting of oligometastatic disease: young age (i.e., <65–70), patient fitness (i.e., KPS ≥70), slow growing cancers (i.e., metachronous vs. synchronous; longer duration to develop metastases) and minimal burden of disease (35). A “wild card” for outcome in these patients may be the primary site (i.e., breast or prostate cancer). Synchronous vs. metachronous development of oligometastases (relative to the primary tumor), was not a significant factor for any outcome in this study, as it was in other studies (36).

Other postulated predictors for oligometastatic characteristics include molecular factors measured both before treatment (37, 38), and in the surveillance period (39). These factors were not considered in our study, but they show promise in predicting outcomes. Post therapy surveillance is especially important since the majority of patient's cancer will progress, as seen in our study (5 year PFS 7.3%). However, roughly two-thirds of patients living >5 years underwent additional local therapy (HSRT/SBRT or surgery) for local recurrence/or for new oligometastatic lesions, and better surveillance can help guide these salvage opportunities.

One limitation of the current study is the variable dose-fractionation schedules used. As described previously (24), fractional doses in excess of 8 Gy were just beginning to be investigated when this study began, and thus the physicians treating patients on this study opted to be somewhat conservative (relative to the SBRT dose-fractionation schedules commonly used today). We attempted to address the discrepancy by converting prescribed dose to BED, and assessed for outcome based on a BED cutoff of 75 Gy (NS). There is compelling evidence for higher doses and lower fractionation (40, 41), though a less aggressive dose-fractionation is seemingly effective (42), and the optimal dose-fraction is unclear. Similarly the PTV expansions in this trial predate improvements in technology that today allow for smaller geometric expansions. Another study limitation is that patients were not randomized, as done in SABR-COMET (11) and other studies. Also, the wide variety in timing of systemic therapy (e.g., before HSRT, after HSRT, and/or after developing widespread metastases) and agents used (with many patients undergoing several different regimens over time) preclude meaningful analysis of the impact of systemic therapy on outcomes. Finally, 74% of patients in our study had only one involved organ, and thus our results may not be generalizable to patients with more extensive oligometastatic or oligoprogressive disease.

In summary, while relatively few patients in the present study have survived >10 years (Figure 1), it is remarkable that non-breast, non-prostate oligometastatic patients have survived for such a long duration. There has been an increasing use of locally aggressive treatments for various oligometastatic primaries in the United States, and further research is needed to help better identify patients most likely to benefit from metastases-directed radiotherapy (43). Recently published randomized trials (11, 13, 14) and ongoing studies will continue to provide additional insight into both survival and control outcomes after SBRT/HSRT for a variety of oligometastatic cancers.

## Data Availability

The datasets generated for this study are available on request to the corresponding author.

## Author Contributions

PO, AK, and MM contributed conception and design of the study. MM and KA organized the database. MM and KA performed the statistical analysis. KA and MM wrote the first draft of the manuscript. KA, AK, DS, PO, and MM wrote sections of the manuscript. All authors contributed to manuscript revision, read and approved the submitted version.

### Conflict of Interest Statement

MM: royalties from UpToDate. PO: research grants from DiaCarta; ownership interest (including patents) and consultant/advisory board member for Entrinsic Health and DiaCarta. The remaining authors declare that the research was conducted in the absence of any commercial or financial relationships that could be construed as a potential conflict of interest.
